# Graph Convolutional Network with Neural Collaborative Filtering for Predicting miRNA-Disease Association

**DOI:** 10.3390/biomedicines13010136

**Published:** 2025-01-08

**Authors:** Jihwan Ha

**Affiliations:** Major of Big Data Convergence, Division of Data Information Science, Pukyong National University, Busan 48513, Republic of Korea; jhha@pknu.ac.kr; Tel.: +82-51-629-4614

**Keywords:** graph convolutional network, neural collaborative filtering, miRNA, disease, machine learning

## Abstract

**Background:** Over the past few decades, micro ribonucleic acids (miRNAs) have been shown to play significant roles in various biological processes, including disease incidence. Therefore, much effort has been devoted to discovering the pivotal roles of miRNAs in disease incidence to understand the underlying pathogenesis of human diseases. However, identifying miRNA–disease associations using biological experiments is inefficient in terms of cost and time. **Methods:** Here, we discuss a novel machine-learning model that effectively predicts disease-related miRNAs using a graph convolutional neural network with neural collaborative filtering (GCNCF). By applying the graph convolutional neural network, we could effectively capture important miRNAs and disease feature vectors present in the network while preserving the network structure. By exploiting neural collaborative filtering, miRNAs and disease feature vectors were effectively learned through matrix factorization and deep learning, and disease-related miRNAs were identified. **Results:** Extensive experimental results based on area under the curve (AUC) scores (0.9216 and 0.9018) demonstrated the superiority of our model over previous models. **Conclusions:** We anticipate that our model could not only serve as an effective tool for predicting disease-related miRNAs but could be employed as a universal computational framework for inferring relationships across biological entities.

## 1. Introduction

MicroRNAs are small non-coding RNAs that are approximately 22 nucleotides in length. By binding to the 3′-untranslated regions (UTRs) of target messenger RNAs (mRNAs) via imperfect base-paring, miRNAs regulate gene expression [1,2,3,4,5]. MicroRNAs play two different roles; while they suppress protein translation by interfering with gene expression, they act as positive regulators [6]. Since the discovery of the first miRNA lin-4, many other miRNAs have been identified using high-throughput techniques [7,8]. Several studies have reported the important roles of miRNAs in various biological processes such as aging [9], apoptosis [10], development [11], and proliferation [12]. Therefore, identifying disease-related miRNAs is of great significance from a molecular biology perspective and for diagnosing complex human diseases. Considering the resources and time required for biological experiments, numerous studies have attempted to develop computational frameworks for detecting disease-related miRNAs.

To date, various studies have assumed that functionally similar miRNAs are highly related to phenotypically similar diseases to predict miRNA–disease associations (MDAs) [13,14].

Ha et al. employed a probabilistic matrix factorization model that integrates miRNA expression as implicit feedback to predict novel MDAs [15]. Additionally, they incorporated disease similarity information into the matrix factorization model, achieving improved performance in identifying MDAs [16]. Furthermore, the authors proposed a groundbreaking model by integrating neural collaborative filtering, based on a deep learning architecture, to infer relationships between miRNAs and diseases [17]. Jiang et al. introduced a novel framework that applies a hypergeometric distribution. To detect disease-related miRNAs, this model utilizes various heterogeneous networks, including miRNAs and diseases [18]. Shi et al. designed a random walk framework to detect disease-related miRNAs by using a bipartite network [19]. Mørk et al. reported an efficient model that utilizes protein associations between miRNAs and diseases [20]. This model gathers protein–miRNA and protein–disease associations via text mining. Xu et al. ranked disease-related miRNAs using disease genes and miRNA–target interaction data [21]. Xuan et al. reported a prediction model for HDMP by exploiting the k-nearest neighbors in a constructed network [22]. The HDMP assumes that miRNAs belonging to the same cluster are strongly associated with the same disease. Chen et al. introduced a prediction method to identify novel MDA (RWRMDA). In the RWRMDA, a random walk algorithm is implemented by constructing a global miRNA functional similarity network [23].

As machine learning is effectively applied in various scientific domains, various models have adopted it to perform research tasks in bioinformatics [24,25,26]. Chen et al. introduced an approach that utilized k-nearest neighbors to infer miRNA–disease associations [27]. This model integrates heterogeneous biological datasets and ranks disease-related miRNAs according to scores assigned by the support vector machine. Chen et al. applied hierarchical agglomerative clustering by considering miRNA–disease bias ratings [28]. This research group proposed a detection method called RLSMDA [29]. RLSMDA uses a semi-supervised classifier to predict miRNAs that are not associated with known diseases. Xiao et al. designed a prediction method that applied non-negative matrix factorization (MF) based on various heterogeneous omics data (GRNMF) [30]. When the recommender algorithm was applied, GRNMF performed well for both miRNAs with no known disease associations and diseases with no known miRNA associations. Li et al. introduced a matrix factorization framework, MCMDA, for disease-related miRNA identification [31]. MCMDA efficiently predicts disease-related miRNAs by updating the adjacency matrix of MDA. Chen et al. developed a method for potential miRNA–disease associations (HGIMDAs), in which various similarity values are used for comprehensive networks [32]. Ha et al. reported a prediction method for PMAMCA that exploits matrix factorization to infer disease-related miRNAs [33]. The authors adopted MF, a machine-learning algorithm that is actively applied in recommendation systems. Using MF, PMAMCA efficiently captured disease-related miRNAs by assigning miRNA expression values to the model. Chen et al. integrated the comprehensive similarity values of miRNA functional similarity, disease semantic similarity, and Gaussian interaction profile kernel similarity to efficiently infer disease-related miRNAs [34]. Chen et al. developed a disease-related miRNA detection method called MDHGI that integrates comprehensive similarity values [35]. Chen et al. developed an NC-MCMDA model to identify novel disease-related miRNAs. NCMCMDA adopts a matrix completion algorithm by combining comprehensive similarity values to reflect similarity-based neighborhood constraints [36]. However, a common drawback of the aforementioned computational models is their high dependence on known miRNA–disease information, and the performance of the models varies greatly depending on parameter selection. Ha et al. leveraged matrix factorization, a commonly employed machine learning approach in recommendation systems, to extract disease-associated miRNAs effectively by incorporating disease and miRNA similarity information [37]. The authors also proposed a machine learning-based model for identifying disease-associated miRNAs by employing metric learning techniques [38]. Ning et al. presented AMHMDA, a novel method integrating attention-aware multi-view similarity networks and hypergraph learning to predict miRNA–disease associations [39]. Jin et al. introduced MAMFGAT, a novel model leveraging adaptive modality fusion and graph attention networks to predict miRNA–disease associations by integrating multiple similarity and association networks [40]. Peng et al. proposed MHCLMDA, a novel method utilizing multiple hypergraph contrastive learning and variational auto-encoders to predict miRNA–disease associations by integrating consistent feature representations across multiple views [41].

Here, we propose a novel and feasible machine learning framework using neural collaborative filtering (NCF) and a graph convolutional network (GCN) to infer miRNA–disease associations (GCNCF). This study aims to (1) overcome the limitations of the inner product score function, which violates the triangle inequality, and (2) build a feasible and effective machine learning approach to infer novel MDA while retaining the network structure and attributes. In this model, we first integrated heterogeneous similarity values such as miRNA functional similarity, disease semantic similarity, and Gaussian interaction profile kernel similarity to construct miRNA and disease similarity networks. Subsequently, a GCN is adopted to capture accurate network embeddings while retaining the structure and properties of the network. Finally, the NCF was applied to overcome the violation of the triangle inequality issue while enhancing the prediction accuracy. Consequently, GCNCF achieved reliable performance in detecting miRNA–disease associations, with AUC scores of 0.9273 and 0.9075 through global and local leave-one-out cross-validation (LOOCV). Moreover, extensive experiments based on evaluation measurements qualitatively confirmed the comparable GCNCF performance.

## 2. Materials and Methods

### 2.1. Human miRNA–Disease Association Data

We collected an MDA dataset from a public online database. HMDD v3.0 is a public database that provides 32281 miRNA–disease associations, including 1102 miRNAs and 850 diseases from 17412 papers [42]. The online database miR2Disease contains information on 3273 miRNA-disease associations for 349 miRNAs and 136 diseases [43]; dbDEMC v2.0 is an online database that provides information on 36 cancer types and 2224 miRNAs [44]. We removed duplicate entries and incorporated disease names via MeSH terms for use as gold standard data [45]. According to the collected miRNA–disease associations, a miRNA–disease association binary matrix was built, defined as follows (Equation (1)):

(1) $$y_{ij}=\left(\begin{array}{c} 1,if\;there\;exists\;relationship\;between\;miRNA\;i\;and\;disease\;j\;\\ 0,\;\;\;\;\;\;\;\;\;\;\;\;\;\;\;\;\;otherwise \end{array}\right)$$

### 2.2. MiRNA Functional Similarity

An edge in a network implies a similarity between two nodes. Therefore, miRNA functional similarity data were obtained through MISIM and employed as edge information for the miRNA functional similarity network (FS) [46]. MISIM provides a pairwise similarity score that reflects the functional relationships between miRNAs based on shared biological pathways, co-regulated genes, and other functional annotations. The FS scores between miRNAs were expressed as *FS*(*m*(*i*),*m(j*)). These scores were directly used to construct the miRNA similarity network, where higher scores indicate stronger functional similarity.

### 2.3. Disease Semantic Similarity

We used a directed acyclic graph (DAG) to estimate similarity values across diseases. DAG is a directed graph with no directed cycle, wherein “directed” refers to the fact that each edge has a defined direction, and “acyclic” denotes the existence of no loop [46]. Using the DAG, we can express the disease *DAG*(*P*) as *(P*, *A*(*P*), *EG*(*P*)). *A(P)* denotes the ancestor nodes of node *P,* and *EG*(*P*) denotes all the edges from the parent node to the child node, which can be calculated as follows (Equations (2) and (3)):

(2) $$DV(P)=\sum_{c\in A(P)}P_{P}(c)$$

(3) $$\left\{\begin{array}{l} P_{P}(c)=1\;\;\;\;\;\;\;\;\;\;\;\;if\;c=P\\ P_{P}(c)=\max\{\Delta_{*}P_{P}(c^{\prime })|c^{\prime }\in children\;of\;c\}\;if\;c\neq P \end{array}\right.$$

In the equations presented, ∆ represents the semantic contribution factor, which quantifies how the semantic value of a disease increases as the distance between two diseases decreases within a semantic framework. This factor reflects the assumption that diseases with closer proximity in a directed acyclic graph (DAG) structure are more likely to share similar characteristics. The scoring system builds on this principle, suggesting that the greater the overlap of elements shared within a DAG, the higher the similarity between the diseases. We define SS as the disease semantic similarity matrix, which provides a quantitative measure of similarity between pairs of diseases. Specifically, the semantic similarity between diseases *i* and *j* is mathematically expressed in Equation (4). This approach enables a systematic and scalable way to assess relationships among diseases based on their semantic context.

(4) $$SS(d(i),d(j))=\frac{\sum_{t\in A(i)\cap A(j)}\left(P_{i}(t)+P_{j}(t)\right)}{DV(i)+DV(j)}$$

### 2.4. Gaussian Interaction Profile Kernel

The Gaussian interaction profile (GIP) kernel is a widely used method in various domains, including genomics, disease analysis, and social network studies, to capture interaction patterns across entities such as genes, diseases, and users [47,48]. In our study, we adopted the GIP kernel to estimate similarity values between miRNAs and diseases, leveraging known miRNA–disease association data as the basis for this calculation. Here, IP(*m*(*i*)) represents the profile vector of miRNA *m*(*i*), indicating its association with a specific disease *d*(*i*). This profile vector effectively captures whether *m*(*i*) is linked to *d*(*i*) through known interactions. Using this information, we computed the GIP similarity (denoted as GS) between two miRNAs, *m*(*i*) and *m*(*j*), based on their respective interaction profiles. The mathematical formulation of GS is provided in Equation (5). By employing the GIP kernel, we ensure that the similarity metric is informed by the global interaction landscape, allowing for a robust and biologically meaningful comparison of miRNA–disease associations.


(5) $$GS(m(i),m(j))=\exp(-r_{l}∥\;IP(m(i))-IP(m(j))\;{∥}^{2})$$

In this context, $r_{m}$ represents the kernel bandwidth, a critical parameter that determines the sensitivity of the Gaussian interaction profile (GIP) kernel. Based on empirical findings from previous studies, we set $r_{m}^{\prime }$ to 1, as this value has been shown to provide reliable and consistent results across various applications. Using this predefined bandwidth, we computed the similarity information between diseases by applying the GIP kernel. This approach ensures that the similarity metric reflects the underlying interaction profiles effectively while maintaining computational efficiency. The detailed formulation of this similarity calculation is presented in Equation (6). By adopting this methodology, we leverage the robustness of the GIP kernel to derive meaningful insights into disease relationships, enabling a systematic evaluation of their associations within the dataset.

(6) $$r_{l}=\frac{r_{d}^{\prime }}{\frac{1}{n_{l}}\sum_{i=1}^{n_{d}}||IP(m(i)|{|}^{2}}$$

### 2.5. Integrated Similarity for miRNAs and Diseases

To construct a comprehensive miRNA similarity network, we integrated functional similarity (FS) information with Gaussian interaction profile (GIP) similarity (GS). This integration combines complementary perspectives, where FS captures biological functionality relationships and GS reflects interaction-based associations. The resulting miRNA similarity value, representing the strength of the connection between miRNAs in the network, was assigned to the edges of the miRNA similarity network. This unified similarity metric was calculated using the formulation presented in Equation (7).

(7) $$\begin{array}{l} S_{m}(m(i),m(j))\\ =\left\{\begin{array}{c} FS(m(i),m(j)),\;if\;m(i)\;and\;m(j)\;have\;functional\;similarity\\ GS_{m}(m(i),m(j),\;\;\;\;\;\;\;\;\;\;\;otherwise \end{array}\right. \end{array}$$

Similarly, comprehensive disease similarity was measured by integrating multiple types of similarity information, combining complementary perspectives to enhance the accuracy and robustness of the analysis. This integrated similarity value captures both functional and interaction-based relationships among diseases. The formulation for calculating the comprehensive disease similarity is provided in Equation (8). This approach ensures that the resulting similarity metric reflects a holistic understanding of disease relationships, enabling more effective network-based analyses and predictions.

(8) $$\begin{array}{l} S_{d}(d(i),d(j))\\ =\left\{\begin{array}{c} SS_{d}(d(i),d(j)),\;if\;d(i)\;and\;d(j)\;have\;semantic\;similarity\\ GS_{d}(d(i),d(j),\;\;\;\;\;\;\;\;\;\;\;otherwise \end{array}\right. \end{array}$$

### 2.6. GCNCF

#### 2.6.1. Node-Level Embeddings via Graph Convolutional Network

**Figure 1:**
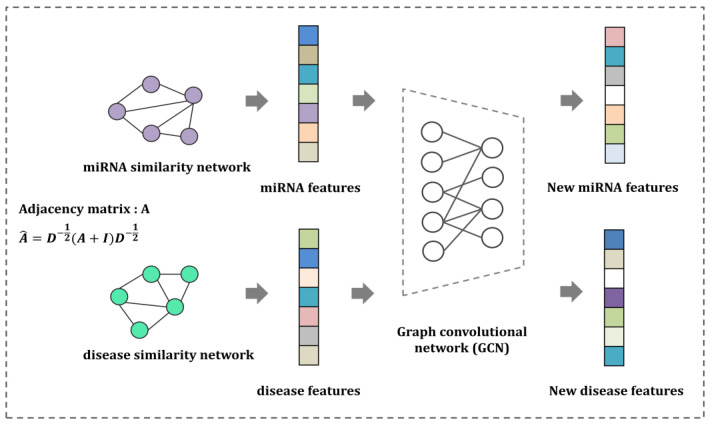
Workflow of node feature embedding. First, we constructed miRNA and disease similarity network through various similarity measuring methods. Next, we applied GCN to calculate new miRNAs and disease node feature embeddings that preserve network structure and properties. The arrows in the diagram indicate the transition from one step to the next in the workflow.

Here, we systemically illustrate the design of an algorithm that can learn low-dimensional node representations while preserving the network topologies and neighboring structures of the nodes [49]. A GCN is a machine learning-based, semi-supervised learning method that is widely used to capture node feature vectors in graph-structured data. The GCN efficiently captures hidden layer representations while conserving both the node features and local graph structure. As shown in Figure 1, two inputs are required to implement the GCN: adjacency matrix A∈$R^{a\;\times \;a}$ and feature matrix F∈$R^{a\;\times \;f}$, where *a* represents the number of nodes present in adjacency matrix A and *f* represents the feature dimensions. The feature matrix F was extracted from the vector information of each miRNA row and disease column in the miRNA–disease association matrix. $A_{v}$ is an edge-weighted similarity miRNA or disease network. Given a miRNA, we stack multiple layers to implement the GCN, which is expressed as follows (Equation (9)):


(9) $$\begin{array}{c} X_{v}^{\left(l+I\right)}=f(X_{v}^{\left(l\right)},-A_{v})\\ =\;\sigma (A_{v}X_{v}^{\left(l\right)}W_{v}^{(l),}) \end{array}$$

*X_v_*^(*l*)^ and *W_v_*^(*l*)^ denote the input and weight matrices in the lth GCN layer, respectively, where σ(·) is the nonlinear activation function. We transformed the adjacency matrix into Equation (10): ${\hat{A}}_{v}$ can be modified to ${\hat{A}}_{v}$ = $A_{v}+I_{v}$, where ${\hat{D}}_{v}$ is the diagonal node matrix of ${\hat{A}}_{v}$ and $I_{v}$ is an identity matrix that contains the significance of nodes through self-loops.

(10) $$X_{v}^{\left(l+1\right)}=\sigma ({\hat{D}}_{v}^{-\frac{1}{2}}{\hat{A}}_{v}{\hat{D}}_{v}^{-\frac{1}{2}}X_{v}^{\left(l\right)}W_{v}^{\left(l\right)})$$

Likewise, the disease-node embedding can be obtained as with disease adjacency matrix $A_{t}\;$ (Equation (11)):

(11) $$Y_{v}^{\left(l+1\right)}=\sigma ({\hat{D}}_{t}^{-\frac{1}{2}}{\hat{A}}_{t}{\hat{D}}_{t}^{-\frac{1}{2}}X_{t}^{\left(l\right)}W_{t}^{\left(l\right)})$$

#### 2.6.2. Generalized Matrix Factorization

The problem encountered in this study was how to accurately predict disease-related miRNAs with the application of linearity of MF and nonlinearity of a multi-layer perceptron (MLP). A common underlying idea of NCF is that we can endow a prediction model with linearity and nonlinearity using a variant of a deep learning model. The original matrix factorization captures the relationship between two objects by considering the inner product of two latent spaces. In this regard, we formulate generalized matrix factorization (GMF) as follows (Equation (12)):

(12) $$f_{1}(m_{u},d_{i})=m_{u}⨀d_{i}$$

where, $m_{u}$ and $d_{i}$ stand for miRNA and disease feature vectors, respectively, which were obtained through GCN, and ⨀ indicated element-wise product function.

(13) $$f^{GMF}=a_{out}(h^{T}\left(\;m_{u}⨀\;d_{i}\right))\;d_{i}$$

Moreover, the meaning of h and $a_{out}$ can be interpreted as the weights of the output layer and activation function, respectively. Setting the activation function to the identity function implies that *h* becomes 1, yielding the same result as the MF (Equation (13)).

#### 2.6.3. Multi-Layer Perceptron

Feature concatenation is widely used in existing multimodal deep-learning architectures [50,51]. However, concatenating vectors alone do not accurately reflect the relationship between two objects. To compensate for this shortcoming, we applied a multilayer perceptron (MLP) to endow the model with nonlinearity by stacking multiple hidden layers. The GCNCF can be expressed with Equation (14).

$$z_{1}=f_{1}\left(m_{u},\;d_{i}\right)=\left[\begin{array}{c} m_{u}\\ d_{i} \end{array}\right],$$

$$f_{2}\left(z_{1}\right)=a_{2}\left(W_{2}^{T}z_{1}+b_{2}\right),$$

…

$$f_{L}\left(z_{L-1}\right)=a_{L}\left(W_{L}^{T}z_{L-1}+b_{L}\right),$$

(14) $$f^{MLP}=\sigma \left({h^{T}f}_{L}(z_{L-1})\right),$$

where $w_{x}$, $a_{x},$ and $b_{x}$ denote the weight matrix, activation function, and bias vector of the xth layer, respectively. Based on the empirical experimental results, the performance of ReLU was observed to be comparable to that of other activation functions, such as the tanh and sigmoid functions. Therefore, the model adopts ReLU as an activation function.

#### 2.6.4. Application of Neural Collaborative Filtering

**Figure 2:**
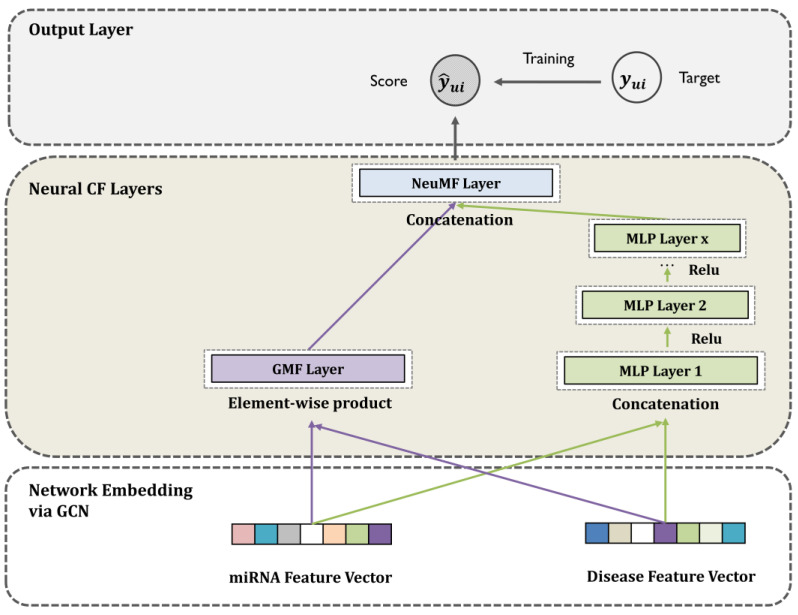
Workflow of neural collaborative filtering for disease-related miRNA extraction. The arrows in the diagram indicate the transition from one step to the next in the workflow.

The most straightforward method for fusing a GMF with an MLP is to share the same miRNA and disease input features. Therefore, we designed a hybrid model that fed the same miRNA and disease feature vectors through a GCN. The GCNCF formulation can be expressed with Equation (15), as follows:

$$f^{GMF}=m_{u}^{G}\cdot d_{i}^{G}$$

$$f^{MLP}=a_{L}(W_{L}^{T}\left(a_{L-1}\left(\ldots a_{2}\left(W_{2}^{T}\left[\begin{array}{c} m_{u}^{M}\\ d_{i}^{M} \end{array}\right]+b_{2}\right)\ldots \right)\right)+b_{L}),$$

(15) $${\overset{´}{y}}_{ui}=(h^{T}\left(\begin{array}{c} f^{GMF}\\ f^{MLP} \end{array}\right))$$

where $m_{u}^{G}$ and $m_{u}^{M}$ denote the network feature embeddings of the GMF and MLP, respectively, and $d_{i}^{G}$ and $d_{i}^{M}$ represent their network feature vectors, GMF and MLP. Figure 2 details the entire process, and Table 1 describes the notations.

## 3. Results

### 3.1. Parameter Settings (Hidden Layers)

The most important factor for an accurate performance evaluation is the variation in performance based on parameter selection. Adam optimizer was used to select the optimal parameter values for our model. Also, to select the model with the best performance, the latter is evaluated by introducing different numbers of hidden layers. As we stack more hidden layers in a deep neural network, we can endow the model with more nonlinearity, which is beneficial for improving performance. We set a series of values for the number of hidden layers and sequentially measured the AUC scores. MLP0 has no hidden layers, whereas MLP5 has five hidden layers. As shown in Table 2, the model with four hidden layers achieved the best performance. Therefore, all the experiments were conducted using a model with four hidden layers.

Several evaluation metrics were employed to evaluate the performance of the GCNCF. LOOCV is widely used to estimate model performance. It is generally divided into two types: global LOOCV considers all diseases simultaneously, whereas local LOOCV considers a specific disease simultaneously. To illustrate the performance of GCNCF clearly, we drew a receiver operating characteristic (ROC) curve, with the X- and Y-axes denoting the true positive rate (TPR) and false positive rate (FPR), respectively. Here, TP and FP refer to properly detected and poorly detected positive samples, respectively, whereas TN and FN indicate correctly identified and poorly predicted negative samples, respectively. We measured TPR and FPR using (Equations (16) and (17)). The AUC score was obtained based on the receiver operating characteristic (AUC) curve, which allowed us to determine the performance of the model.

(16) $$FPR=\frac{FP}{FP+\;TN}$$

(17) $$TPR=\frac{TP}{TP+FN}$$

Additionally, the values under the PR curve (AUPR) were calculated based on precision and recall, which can be measured using Equations (18) and (19). A PR curve is often used when the classes are imbalanced. Additionally, extensive evaluation metrics such as accuracy (ACC) and Matthew’s correlation coefficient (MCC) were measured using Equations (20) and (21) to measure performance more accurately.

(18) $$precision=\frac{TP}{TP+\;FP}$$

(19) $$recall=\frac{TP}{TP+FN}$$

(20) $$ACC=\frac{TP+TN}{TP+TN+FP+FN}$$

(21) $$MCC=\frac{TP\times TN-FP\times FN}{\sqrt{\left(TP+FP)(TP+FN)(TN+FP)(TN+FN\right)}}$$

### 3.2. Performance Evaluation with Existing Approaches

**Figure 3:**
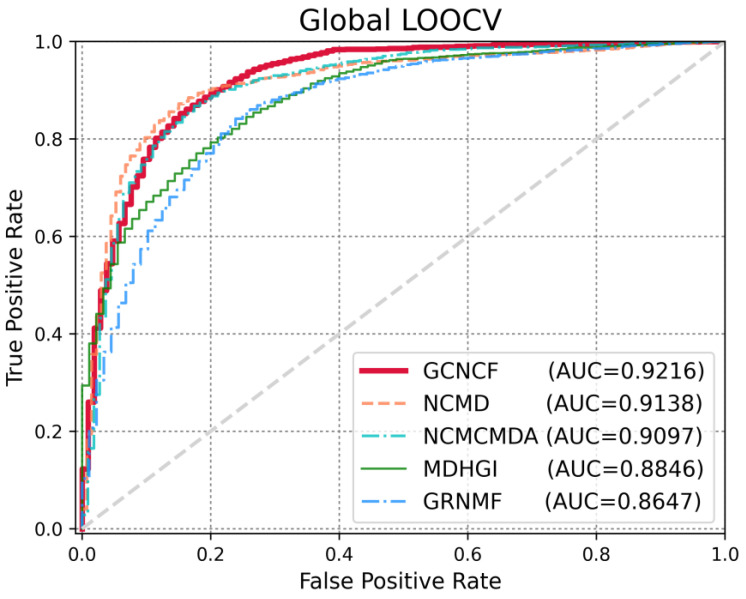
Comparison of model performance based on global LOOCV.

**Figure 4:**
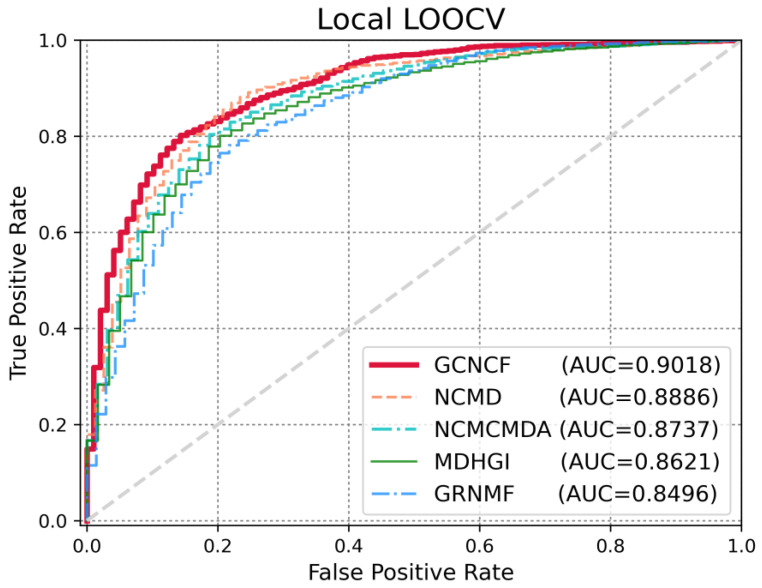
Comparison of model performance based on local LOOCV.

To accurately measure the performance of GCNCF, we conducted a comparative experiment with the latest studies, namely, NCMD [17], NCMCMDA [36], MDHGI [35], and GRNMF [30]. Based on the global LOOCV (Figure 3), GCNCF achieved a meaningful AUC score of 0.9216, which was superior to those of NCMD (0.9138), NCMCMDA (0.9097), MDHGI (0.8846), and GRNMF (0.8647). We used local LOOCV to demonstrate the performance of GCNCF in identifying novel disease-related miRNAs. As shown in Figure 4, the GCNCF obtained an AUC score of 0.9018, indicating that it performed better than NCMD (0.8886) and NCMCMDA (0. 8737), MDHGI (0.8621), and GRNMF (0.8496). Moreover, the performance of the model was measured quantitatively using AUPR, ACC, and MCC. As summarized in Table 3 and Table 4, we proved that GCNCF performed better based on various statistical values.

### 3.3. Ablation Study

**Figure 5:**
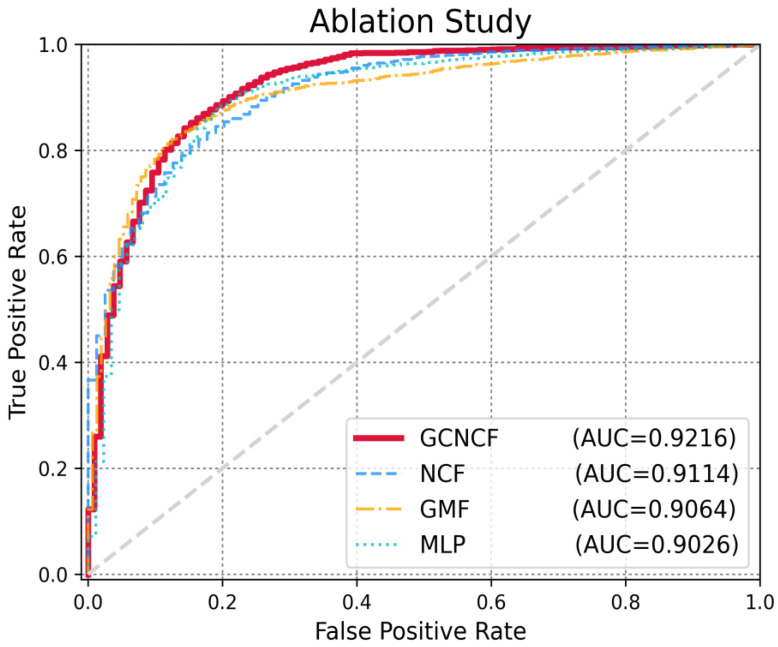
Performance evaluation in the ablation study. GCNCF with GMF, MLP, and GCN modules achieved superior performance.

The most straightforward way to accurately evaluate each machine-learning model component is to implement an ablation study. An ablation study is an experiment in which the components of a machine learning model are removed to assess the effect of the removed components on model performance. In this study, we propose a hybrid model that combines the GMF and MLP to ascertain the benefits of linearity and nonlinearity. To evaluate the performance of the GMF and MLP components, we removed the corresponding components from the model and evaluated their performances. The GMF model excludes the MLP module from the proposed GCNCF model, whereas the MLP model excludes the GMF module from the GCNCF model. One of the main contributions of this study is the proposed method of effectively capturing high-dimensional structural information from the input graph using graph convolutional networks (GCNs). This is particularly useful for modeling complex relationships between entities, especially in graph-based data, where interactions can be more accurately represented. To validate this approach, we conducted experiments comparing the performance of the model with and without the use of GCN. In our framework, GCN serves solely as a feature extractor and does not function as an independent predictor or classifier. In other words, GCN’s role is limited to extracting informative feature vectors from the input graph, which are then fed into the neural collaborative filtering (NCF) model for prediction. Therefore, using GCN alone does not yield meaningful predictive results, and we define the model without GCN as the ’NCF model’. The experimental results show that when GCN is used to extract feature vectors, the performance of the model significantly outperforms the NCF model that does not utilize GCN. This demonstrates that GCN is highly effective at extracting high-quality feature representations from the graph, which plays a crucial role in improving the predictive performance of the model. The results confirm that GCN is not only effective in processing graph data but also essential for optimizing model performance by providing rich feature vectors that enhance the overall accuracy of predictions. As illustrated in Figure 5, the model with the GMF, MLP, and GCN components (GCNCF) performed better than when each component was used alone.

### 3.4. Case Studies

To further demonstrate the GCNCF performance, we examined two major human cancers. Breast cancer (BC) is common in women and has a high fatality rate worldwide [52]. Various studies have shown that miRNAs are biological factors that significantly influence BC development. For example, miR-202 and miR-718 are highly expressed in patients with BC, indicating their significant roles as biomarkers for early BC [53]. Therefore, we performed a case study to determine whether the candidates predicted by the GCNCF were related to BC. Table 5 shows that all top-ranked candidates were related to BC using the gold standard dataset.

We conducted additional experiments on lung cancer (LC). LC is a malignant lung tumor and a major cause of cancer-related mortality [54]. Smoking tobacco is the primary cause of LC. Additionally, miRNAs play important roles in LC development [55]. Therefore, the top 50 candidates were ranked according to the predicted values obtained by the GCNCF. Consequently, all miRNAs were shown to be related to LC based on the gold-standard datasets (Table 6). Based on the experimental results, we demonstrated that GCNCF exhibited excellent performance in identifying disease biomarkers.

### 3.5. Pathway Analysis

**Figure 6:**
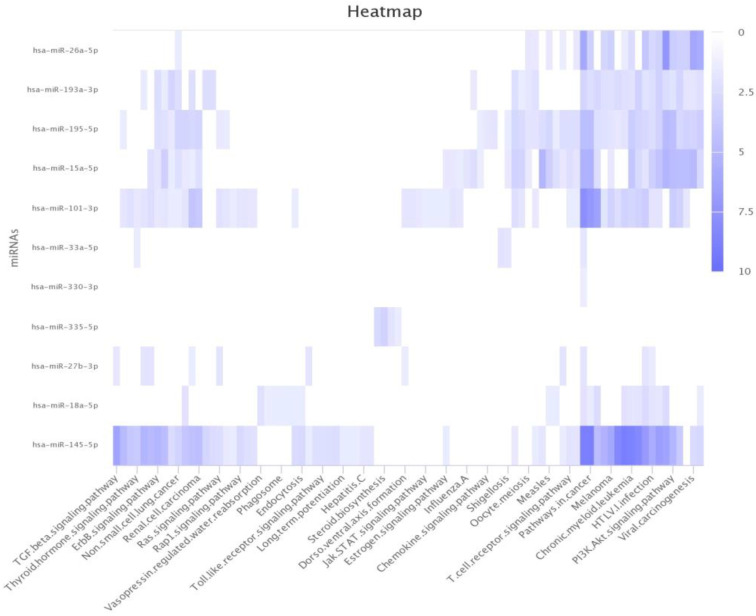
Heatmap illustration based on lung cancer-related miRNAs.

Pathway analysis is a functional enrichment analysis method that provides information on the molecular interactions and underlying biology of differentially expressed genes and proteins. Our model was expected to predict disease-related miRNAs and provide clues for inferring miRNA-associated activities. Therefore, we performed a pathway analysis according to their targets. DIANA-miRPath v3.0, a web tool, provides information on controlled pathways and miRNA regulatory roles [56]. We used DIANA-miRPath v3.0, to evaluate the underlying biological roles of LC-related miRNA candidates. As shown in Table 7, most functions were associated with LC-related pathways. The Hippo signaling pathway regulates cell proliferation and death, which substantially affects LC incidence [57]. Hippo signaling plays a key role in lung disease and modulates various cellular functions [58]. Furthermore, we applied mirPathDB 2.0 to intuitively express the relationship between the miRNA target and the pathway using a heatmap [59]. The dark color in the heatmap indicates that the miRNA target and the corresponding pathway are closely related (Figure 6). Based on the experimental results, the excellent performance of the GCNCF in the extraction of disease-related miRNAs was validated.

## 4. Discussion and Conclusions

With the exponential growth in heterogeneous networks, the extraction of meaningful network embeddings via machine learning has become increasingly important. The crucial factors for network embedding are (1) learning low-dimensional representations for nodes in the network and (2) capturing the semantics behind the pairwise relationship (e.g., miRNA–disease association prediction). This study was motivated by the fact that the network implies node structural roles and attributes while learning the low-dimensional representations of nodes. Specifically, this study addresses the MDA prediction problem by formalizing neural collaborative filtering using GCNCF. GCNCF comprehensively integrates miRNA and disease similarities through miRNA functional similarity, disease semantic similarity, and a Gaussian interaction profile kernel to build miRNA and disease networks. We focused on modeling a network-embedding approach with a GCN that was suitable for learning low-dimensional vectors while aiming to capture the network structure and roles more accurately and realistically. Consequently, we systemically fused the linearities and non-linearities of miRNAs and disease feature vectors by designing neural collaborative filtering to predict novel miRNA–disease associations. The experimental results under various evaluation metrics validated that GCNCF outperformed the four previous models.

GCNCF leverages graph convolutional networks (GCNs) to capture higher-order structural information from the input graph, effectively modeling complex relationships between entities. Unlike NCMD and MDHGI, which primarily rely on matrix decomposition and feature aggregation, GCNCF dynamically updates node representations through neighborhood information propagation. Additionally, the integration of neural collaborative filtering (NCF) enables the modeling of nonlinear and latent interactions often overlooked by traditional matrix factorization methods like GRNMF. This synergistic combination of GCN and NCF provides dual benefits: GCN establishes a robust foundation for representation learning, while NCF enhances predictive accuracy by capturing intricate interaction dynamics. These advancements are validated by the experimental results, where GCNCF consistently outperforms referenced models across multiple evaluation metrics, as detailed in the Results section. We believe these enhancements clearly highlight the novel contributions of GCNCF and its advantages over existing approaches.

However, our model has room for further improvement, which could be explored in future work. The model can be broadly divided into two stages: the feature representation stage, where diseases and miRNAs are mapped to low-dimensional vectors, and the prediction stage, where the relationships between miRNAs and diseases are inferred. To improve the first stage, employing more advanced machine learning models for feature representation could provide more accurate and meaningful representations of these objects, making them more comprehensible to the computer. In addition, applying more sophisticated machine learning techniques in the relationship prediction model could enhance performance and offer significant potential for model improvement. Also, generalizing the model to new, unseen diseases remains challenging. Exploring transfer learning or integrating domain-specific knowledge could help improve performance for diseases with limited data.

## Figures and Tables

**Table 1:** Notations.

Symbol	Description
A	Adjacency matrix
F	Feature matrix
W	Weight matrix
D	Diagonal matrix of A
I	Identity matrix

**Table 2:** Performance evaluation by differentiating the number of hidden layers.

	MLP0	MLP1	MLP2	MLP3	MLP4	MLP5
AUC	0.8658	0.8814	0.8957	0.9183	0.9216	0.9074

**Table 3:** Performance comparison based on various evaluation metrics (global LOOCV).

Method	AUC	AUPR	ACC	MCC
GCNCF	0.9216	0.9124	0.9006	0.7819
NCMD	0.9138	0.8602	0.8734	0.7514
NCMCMDA	0.9097	0.8678	0.8613	0.7492
MDHGI	0.8846	0.8281	0.8265	0.7252
GRNMF	0.8647	0.8365	0.8209	0.7216

**Table 4:** Performance comparison based on various evaluation metrics (local LOOCV).

Method	AUC	AUPR	ACC	MCC
GCNCF	0.9018	0.8931	0.8862	0.7547
NCMD	0.8886	0.8468	0.8504	0.7269
NCMCMDA	0.8737	0.8562	0.8372	0.7206
MDHGI	0.8621	0.8117	0.8062	0.7186
GRNMF	0.8496	0.8323	0.7876	0.6988

**Table 5:** Top 50 miRNA candidates observed to be related to breast cancer through GCNCF.

Rank	Name	Evidence	Rank	Name	Evidence
1	hsa-mir-493-5p	dbDEMC	26	hsa-mir-19b	hmdd, dbDEMC
2	hsa-mir-6510-3p	dbDEMC	27	hsa-mir-1322	dbDEMC
3	hsa-mir-920	dbDEMC	28	hsa-mir-297	dbDEMC
4	hsa-mir-590-5p	dbDEMC	29	hsa-mir-766-3p	dbDEMC
5	hsa-mir-744	dbDEMC	30	hsa-mir-485-5p	dbDEMC
6	hsa-mir-604	dbDEMC	31	hsa-mir-365b	hmdd
7	hsa-mir-500	dbDEMC	32	hsa-mir-523	dbDEMC
8	hsa-mir-4306	hmdd, dbDEMC	33	hsa-mir-26b	hmdd, dbDEMC
9	hsa-mir-520b	hmdd, dbDEMC	34	hsa-mir-205	hmdd, miR2Disease, dbDEMC
10	hsa-mir-4662a-5p	dbDEMC	35	hsa-mir-515-2	hmdd
11	hsa-mir-371	dbDEMC	36	hsa-mir-3614-5p	dbDEMC
12	hsa-mir-1224-3p	dbDEMC	37	hsa-mir-409-3p	dbDEMC
13	hsa-mir-3150b-3p	dbDEMC	38	hsa-mir-183-3p	dbDEMC
14	hsa-mir-21-3p	dbDEMC	39	hsa-let-7a-1	hmdd
15	hsa-mir-200c	hmdd, miR2Disease, dbDEMC	40	hsa-mir-652	hmdd
16	hsa-mir-219-2-3p	dbDEMC	41	hsa-mir-548q	dbDEMC
17	hsa-mir-106a	hmdd, dbDEMC	42	hsa-mir-192-3p	dbDEMC
18	hsa-mir-1256	dbDEMC	43	hsa-mir-503-5p	dbDEMC
19	hsa-mir-1277-3p	dbDEMC	44	hsa-mir-1238	dbDEMC
20	hsa-mir-1306-5p	dbDEMC	45	hsa-mir-1286	dbDEMC
21	hsa-mir-1-2	hmdd	46	hsa-mir-517c	dbDEMC
22	hsa-mir-205-5p	dbDEMC	47	hsa-mir-551b-3p	dbDEMC
23	hsa-mir-155	hmdd, miR2Disease, dbDEMC	48	hsa-mir-203a	hmdd, dbDEMC
24	hsa-mir-362-3p	dbDEMC	49	hsa-mir-663b	dbDEMC
25	hsa-mir-130b-3p	dbDEMC	50	hsa-mir-653	dbDEMC

**Table 6:** Top 50 miRNA candidates observed to be related to lung cancer through GCNCF.

Rank	Name	Evidence	Rank	Name	Evidence
1	hsa-mir-31-5p	dbDEMC	26	hsa-mir-181a-2-3p	dbDEMC
2	hsa-mir-758-3p	dbDEMC	27	hsa-mir-370	dbDEMC
3	hsa-mir-199a-3p	dbDEMC	28	hsa-mir-1303	dbDEMC
4	hsa-mir-29b-3p	dbDEMC	29	hsa-mir-224	hmdd, miR2Disease, dbDEMC
5	hsa-mir-1306-3p	dbDEMC	30	hsa-mir-34b-3p	dbDEMC
6	hsa-mir-625	dbDEMC	31	hsa-mir-629	hmdd, dbDEMC
7	hsa-mir-193a-3p	dbDEMC	32	hsa-mir-15b	dbDEMC
8	hsa-mir-299-5p	dbDEMC	33	hsa-mir-500b-5p	dbDEMC
9	hsa-mir-1249-3p	dbDEMC	34	hsa-mir-7-3	hmdd
10	hsa-mir-513-5p	dbDEMC	35	hsa-mir-28	dbDEMC
11	hsa-mir-493-3p	dbDEMC	36	hsa-mir-205	hmdd, miR2Disease, dbDEMC
12	hsa-mir-145-5p	dbDEMC	37	hsa-mir-527	dbDEMC
13	hsa-mir-3917	dbDEMC	38	hsa-mir-2110	dbDEMC
14	hsa-mir-1976	dbDEMC	39	hsa-mir-378	hmdd, dbDEMC
15	hsa-mir-93-3p	dbDEMC	40	hsa-mir-1201	dbDEMC
16	hsa-mir-363	dbDEMC	41	hsa-mir-381	hmdd, dbDEMC
17	hsa-mir-144-5p	dbDEMC	42	hsa-mir-645	dbDEMC
18	hsa-mir-196a-5p	dbDEMC	43	hsa-mir-20a-3p	dbDEMC
19	hsa-mir-874-3p	dbDEMC	44	hsa-mir-9-1	hmdd
20	hsa-mir-1245	hmdd, dbDEMC	45	hsa-mir-4444	dbDEMC
21	hsa-mir-133a	hmdd, dbDEMC	46	hsa-mir-599	dbDEMC
22	hsa-mir-148b	hmdd, dbDEMC	47	hsa-mir-215	hmdd, dbDEMC
23	hsa-mir-7702	dbDEMC	48	hsa-mir-4449	dbDEMC
24	hsa-mir-3684	dbDEMC	49	hsa-let-7c-3p	dbDEMC
25	hsa-mir-141-3p	dbDEMC	50	hsa-mir-192	hmdd, miR2Disease, dbDEMC

**Table 7:** Enrichment results for lung cancer-related miRNAs.

KEGG Pathway	*p*-Value
Hippo signaling pathway	4.02488531822 × 10^−11^
TGF-beta signaling pathway	4.91330975548 × 10^−7^
Chronic myeloid leukemia	0.00011125247798
ECM-receptor interaction	0.000162660167095
Pathways in cancer	0.000262647049558
Thyroid hormone signaling pathway	0.000425690925118
Transcriptional misregulation in cancer	0.000611500467761
FoxO signaling pathway	0.00338511556088
Renal cell carcinoma	0.00375870022186
Pancreatic cancer	0.0182653781647
Small cell lung cancer	0.0318225482279

## Data Availability

Data derived from public domain resources [42].
